# Hypertensive disorders of in-vitro fertilization pregnancies: A study from Kosovo

**Published:** 2018-02

**Authors:** Merita Vuniqi-Krasniqi, Myrvete Paçarada, Qëndresë Daka, Zeqir Dervishi, Astrit Bimbashi, Kushtrim Dakaj

**Affiliations:** 1 *Department of Gynecology, University Clinical Center of Kosova, Medical Faculty, University of Pristine, Prishtine, Kosovo.*; 2 *Department of Pathophysiology, University Clinical Center of Kosova, Medical Faculty, University of Pristine, Pristine, Kosovo.*; 3 *University Hospital of Obstetrics and Gynecology Koço Gliozheni, University of Medicine, Tirana, Albania.*; 4 *Dentistry School, Medical Faculty, University of Pristine, Pristine, Kosovo.*

**Keywords:** In vitro fertilization, Gestational hypertension, Outcomes, Kosovo

## Abstract

**Background:**

Relationships between in-vitro fertilization (IVF), gestational hypertension, and pregnancy outcomes are demonstrated in a number of studies. However, it is still debated if IVF treatment or specific characteristics of infertile patients are responsible for worse obstetrical and neonatal outcomes.

**Objective:**

The aim of this study was to investigate maternal characteristics associated with hypertensive disorders (HD) in IVF conceived pregnancies and to assess the obstetrical and neonatal outcomes.

**Materials and Methods:**

In this observational, cross-sectional study, 207 pregnant women who underwent IVF treatment were consecutively divided into two groups: a group that had no HD during pregnancy (IVF group) and a group that had HD during pregnancy (IVF+HD group). Maternal, obstetrical and neonatal data of the two groups were compared.

**Results:**

Some maternal characteristics were significantly higher in IVF+HD compared to IVF group such as: older age (p=0.0001), primiparity (p=0.038), obesity (p=0.0001), and cigarette smoking (p=0.0001). There were no significant differences between the groups in regard to obstetrical outcomes besides premature rupture of membranes time that was significantly higher in IVF+HD group compared to IVF group (p=0.036). In regard to neonatal outcomes, the only statistically significant difference was in the 5^th^ min Apgar score, which was higher in IVF+HD group compared to IVF group without HD (p=0.002).

**Conclusion:**

With regard to significantly higher differences in maternal characteristics of IVF conceived pregnancies complicated with HD, compared to uncomplicated ones, development of a specific national prevention measure for HD of IVF conceived pregnancies in Kosovo is strongly suggested. In addition, setting up of a national registry is recommended in order to evaluate the outcomes of IVF treatments properly.

## Introduction

The in-vitro fertilization (IVF) treatment is considered a routine medical practice in the management of infertility (1). Since the introduction in 1978, the technique has evolved and indications have been expanded. Today, the number of children born worldwide using IVF treatment exceeds 5 million (2). Although the IVF success rate has been increased, especially over the past decade, data regarding obstetrical and neonatal outcomes of IVF treatments remain controversial. Data from observational studies indicate that complications more associated with IVF treatments include: multiple births, ectopic pregnancy, maternal hemorrhage, gestational diabetes, hypertensive disorders, placenta previa, placental abruption, delivery by C-section, preterm birth, low birth weight (LBW) and very LBW infants, congenital anomalies and increased perinatal morbidity and mortality (3-7).

Data from population-based studies point out that up to 10% of all pregnancies are complicated by hypertensive disorders (HD) embracing: chronic hypertension (high blood pressure presented before pregnancy or before 20 wk of gestation); gestational hypertension (transient hypertension of pregnancy or chronic hypertension presented after 20 wk of gestation); preeclampsia (gestational hypertension and proteinuria, with or without pathological edema); or eclampsia (preeclampsia associated with seizures that cannot be attributable to other causes) (8-11). Among them, gestational hypertension is most frequently presented 5-6%, followed by preeclampsia-eclampsia 2-3% and chronic hypertension 1%, whereas preeclampsia remains the leading cause of maternal and perinatal morbidity and mortality worldwide (12-14). These disorders are also associated with adverse pregnancy outcomes such as C-section, placental abruption, fetal growth restriction and demise, preterm delivery, LBW, neonatal morbidities or perinatal death (15-17). 

Many studies have been conducted to study relationships between IVF, HD during pregnancy and pregnancy outcomes, however, still many questions remain unclear. In contrast to developed countries, IVF treatments were not performed in Kosovo until 2010 when the first private clinic that offered IVF treatment was opened. The aim of this study was to investigate maternal characteristics associated with HD in IVF conceived pregnancies and to assess the obstetrical and neonatal outcomes.

## Materials and methods

This observational, cross-sectional study was reported in line with the format and methods as suggested by STROBE guidelines (18). The study was conducted during a 2 yr period, from January 2014-December 2015. 

All pregnant women were considered eligible to be included if they conceived through IVF treatment and were at least of 20 wk gestation. Pregnant women were categorized in two groups: a group of pregnant women that conceived through IVF treatment and had no HD during pregnancy (IVF group) and a group of pregnant women that conceived through IVF treatment and had HD during pregnancy (IVF+HD group). Hypertensive disorders in pregnancy were diagnosed based on the diagnostic criteria set by the National High Blood Pressure Education Program Working Group (8). 

Women with previous chronic hypertension were not included in the study. Maternal characteristics were recorded, in data collection forms, by a nurse at the first time when patients showed up, while gynecologist continuously obtained obstetric and neonatal data including: cervical cerclage, premature rupture of membranes, C-section, birth weight, gestational age, preterm birth, Apgar score, and perinatal morbidity. Data were collected and checked for completeness before entered into a personal computer.

**Ethical consideration**

The study protocol was reviewed and approved by the ethics committee of the Medical Faculty of University of Prishtina (Ref. number: 10389 dt09/12/2014), while informed consent was obtained from all patients.

**Statistical analysis**

Maternal, obstetrical and neonatal data of IVF conceived pregnancies complicated with HD were compared to the uncomplicated ones using chi-squared or Fisher’s exact tests for categorical variables and students’ *t*-tests for continuous variables. Categorical variables are expressed as numbers and/or percentages, while continuous variables as the mean±SD. Statistical analysis was performed using Statistical Package for the Social Sciences, version 22.0, SPSS Inc, Chicago, Illinois, USA (SPSS). Values of p<0.05 were considered significant and p<0.001 as highly significant.

## Results

Main maternal characteristics of our study population are presented in table I. Hypertensive disorders were presented in 54 cases (36 cases with gestational hypertension, 15 cases with preeclampsia and three cases with eclampsia, while no women showed signs and symptoms of hemolysis, elevated liver enzyme levels, and low platelet levels (HELLP) syndrome). The difference in the mean age of women between two groups was statistically highly significant (35.0±5.5 yr IVF group vs. 39.7±7.8 yr IVF+HD group, p=0.0001). The percentage of mothers above 35 yr of age was 79.6 % in the IVF+HD group compared to 47.7 % in the IVF group (p=0.0001). There was no difference between the two groups in regard to working conditions. The majority of women in both groups were housewives 74%. 

We noted a statistical significance between two groups in regard to parity, for 9.3% of women in the IVF+HD group it was their third or more pregnancy compared to 2.0% of women in IVF group (p=0.038). However, we noted no difference between the two groups when compared to the infertility type and multiple pregnancies. The mean duration of primary infertility in all participants was 9.9±5.2 yr, while for secondary infertility the duration was 6.7±4.0 yr. Although the proportion of multiple pregnancies was higher in the IVF+HD group, the difference was not statistically significant (42.6% IVF+HD group vs. 38.5% IVF group, p=0.79). 

Women in the IVF+HD group were more likely to be obese (77.8% vs. 15.0%, p=0.0001) and to smoke (87.0% vs. 13.1%, p=0.0001) than those in the IVF group, whereas all women declared that they do not consume alcohol. The prevalence of diabetes mellitus was not significantly different between the two groups (5.6% among IVF+HD group vs. 1.3% among IVF group, p=0.11). 

Table II depicts the obstetrical and neonatal outcomes of the study population. The frequency of various complications between two groups varied but, the difference was not statistically significant. A total of 18 women, from both groups, underwent cervical cerclage (14.8% in IVF+HD group vs. 6.5% in IVF group, p=0.088). The rate of premature rupture of membranes (PROM) was 13.7% in IVF group compared to 7.4% in IVF+HD group (p=0.330). In regard to PROM time, the difference was statistically significant 5.5±4.9 hr in IVF+HD group vs. 8.8±11.1 hr in IVF group (p=0.036). The percentage of women who delivered by Caesarean section was very high 86.0% (92.6% in IVF+HD group vs. 83.7% in IVF group, p=0.116).

A total of 75 (35 F/40 M) infants in the IVF+HD group and 216 (109 F/107M) infants in the IVF group were born. There was no statistically significant difference between the two groups of babies in regards to their birth weight and gestational age. The mean birth weight difference of the babies was 197 g (2644±722.7 g in IVF+HD group vs. 2447±770.5 g in IVF group, p=0.056), whereas the difference in gestational age was 0.3 wk (36.1±2.2 wk in IVF+HD group vs. 35.8±3.2 wk in IVF group, p=0.524). 1^st^ and 5^th^ min Apgar scores of babies delivered in the IVF+HD group were, in general, higher compared to the IVF group. However, the difference was statistically significant only for the 5th min Apgar score (8.4±0.8 scores in IVF+HD group vs. 7.9±1.3 scores in IVF group, p=0.002) and not for the 1st min (7.0±1.7 scores in IVF+HD group vs. 6.6±1.8 scores in IVF group, p=0.093). 

In total, 49.3 % of babies were born preterm (53.7% in IVF+HD group vs. 47.7% in IVF group, p=0.54), while 6.8% of babies were diagnosed with intrauterine growth restriction (11.1% in IVF+HD group vs. 5.2% in IVF group, p=0.20) during pregnancy. The difference of the neonatal death was not statistically significant (3.7 % in IVF+HD group vs. 5.2% in IVF group, p=0.999).

**Table I.** Main maternal characteristics of study population

**Maternal characteristics**		**IVF+HD group** * ** (n=54)**	**IVF group** ** ** (n=153)**	**p-value**
Age (yr)		39.7 ± 7.8	35.0 ± 5.5	
	Range <35	11 (20.4)	80 (52.3)	0.0001^b^
	Range ≥35	43 (79.6)	73 (47.7)	
Work				
	Housewife	40 (74.1)	114 (74.5)	0.906
	Worker	14 (25.9)	36 (23.5)	
	Student	-	3 (2.0)	
Parity				
	I	47 (87.0)	138 (90.2)	0.038^a^
	II	2 (3.7)	12 (7.8)	
	III+	5 (9.3)	3 (2.0)	
Multiple pregnancy				
	Singleton	31 (57.4)	94 (61.4)	0.79
	Twins	22 (40.7)	55 (35.9)	
	Triplets	1 (1.9)	4 (2.6)	
Primary Infertility		34 (63)	92 (60.1)	0.838
Primary Infertility (yr)		10.5 ± 6.5	9.8 ± 4.6	0.392
Secondary Infertility		4 (7.4)	6 (3.9)	0.29
Secondary Infertility (yr)		7.5 ± 6.4	6.25 ± 3.5	0.076
Obesity		42 (77.8)	23 (15.0)	0.0001^b^
Smoking		47 (87.0)	20 (13.1)	0.0001^b^
Diabetes mellitus		3 (5.6)	2 (1.3)	0.11

Data are presented as mean±SD or as n (%).

Continuous variables evaluated by independent students’* t*-test, categorical variables evaluated by Chi-squared or Fisher's exact test.

Values of p<0.05

* had not hypertensive disorders (HD) during pregnancy

** had HD during pregnancy

a were considered significant and p<0.001

b as highly significant.

**Table II:** Obstetrical and neonatal outcomes of study population

**Obstetrical outcomes**	**IVF+HD group** * ** (n=54)**	**IVF group** ** ** (n=153)**	**p-value**
Caesarean section	50 (92.6)	128 (83.7)	0.116
Cervical cerclage	8 (14.8)	10 (6.5)	0.088
PROM	4 (7.4)	21 (13.7)	0.330
PROM (time )	5.5 ± 4.9	8.8 ± 11.1	0.036^a^
**Neonatal outcomes**	**75 (35 F/40M)**	**216 (109 F/107 M)**	**p-value**
Birth weight (gr)	2644 ± 722.7	2447 ± 770.5	0.056
Gestational age (wk)	36.1 ± 2.2	35.8 ± 3.2	0.524
Preterm births	29 (53.7)	73 (47.7)	0.54
IUGR	6 (11.1)	8 (5.2)	0.20
Apgar1 score	7. 0 ± 1.7	6.6 ± 1.8	0.093
Apgar5 score	8.4 ± 0.8	7.9 ± 1.3	0.002^a^
Neonatal death	2 (3.7)	8 (5.2)	0.999

Continuous variables evaluated by independent students’ *t*-test, categorical variables evaluated by chi-squared or Fisher's exact test.

Data are presented as mean±SD or as n (%).

Values of p<0.05

PROM: Premature rupture of membranes

IUGR: Intrauterine growth restriction

F: Female

M: Male.

* had not hypertensive disorders (HD) during pregnancy

a were considered significant.

** had HD during pregnancy

## Discussion

Little is known about the maternal characteristics associated with HD in IVF conceived pregnancies and their obstetrical and neonatal outcomes in Kosovo. In the present study, we compared maternal, obstetrical and neonatal data of IVF conceived pregnancies complicated with HD to uncomplicated ones.

We found that, in Kosovo, hypertensive disorders were presented among 26.1% of women that conceived through IVF treatment. Data from this study highlight that some maternal characteristics were significantly higher in IVF conceived pregnancies complicated with HD compared to uncomplicated ones including older age (p=0.0001), primiparity (p=0.038), obesity (p=0.0001) and cigarette smoking (p=0.0001). Advanced maternal age, primiparity, obesity, and smoking are all well-known risk factors for HD in pregnancy, in addition to IVF treatment, and our findings are in line with the literature reports (19). On the contrary, in our study, multiple pregnancies and diabetes mellitus do not appear as important risk factors for HD complications in IVF conceived pregnancies, as the difference was not statistically significant in between the two groups (p=0.79, p=0.11 respectively). Although, data from other countries show that the majority of women who seek for IVF treatments have a higher education attainment level and household income, the case was different in Kosovo. Furthermore, we found no difference in regard to HD occurrence, that in many studies are linked to lower education level, as the majority of 74% of women in both groups were housewives. However, we think that our results could have been influenced by the high unemployment rate in our country, additionally to the literacy rate of our population (20, 21). The differences in obstetrical outcomes were not significant between the two groups of IVF conceived pregnancies, although, both cervical cerclage and PROM tended to occur more among IVF pregnancies that were complicated with HD. However, PROM time was significantly higher in IVF pregnancies that were complicated with HD compared to uncomplicated ones (p=0.036). In comparison with data from other countries, the percentage of women who needed to be delivered by Caesarean section, among both groups, was very higher (86.0%) (22, 23). 

In regard to neonatal outcomes, in general, neonates of women which pregnancies were complicated by HD had higher birth weight (197g); longer gestational age (0.3 wk); and higher 1st and 5th min Apgar score outcomes (0.3 scores and 0.4 scores, respectively), but the only statistically significant difference was the 5th min Apgar score (p=0.002). Whereas, in contrary, it was noted a higher prevalence of intrauterine growth restriction (5.9%) and preterm births (6.0%) among them.

To our knowledge, this is the first study that evaluated maternal characteristics, obstetrical and neonatal outcomes of IVF pregnancies in Kosovo. We have to acknowledge that due to the cross-sectional design of the study, our results could be prone to limitations. Therefore, it is recommended that more prospective studies needed to be performed in order to obtain more accurate results.

## Conclusion

Our study provides that, in Kosovo, IVF pregnancies complicated with HD were more common among women of older age, primipara, obese and cigarette smokers. Regarding the increased rate of infertility in Kosovo, it is predicted to have an increase in the number of women that will conceive through IVF procedures. These findings could serve to the development of specific national prevention measure for HD of IVF conceived pregnancies. Setting up of a national registry of assisted conception is recommended in order to properly evaluate the effectiveness of IVF treatment and the outcomes of women and their children.

## Note

***This article extracted from Ph.D. thesis. (Merita Vuniqi-Krasniqi*** *)*

## References

[1] Marianowski P, Dąbrowski FA, Zyguła A, Wielgoś M, Szymusik I (2016). Do we pay enough attention to culture conditions in context of perinatal outcome after in vitro fertilization? Up-to-date literature review. Biomed Res Int.

[2] van Loendersloot L, Repping S, Bossuyt PM, van der Veen F, van Wely M (2014). Prediction models in in vitro fertilization; where are we? A mini review. J Adv Res.

[3] Mukhopadhaya N, Arulkumaran S (2007). Reproductive outcomes after in-vitro fertilization. Curr Opin Obstet Gynecol.

[4] Romundstad LB, Romundstad PR, Sunde A, von Düring V, Skjaerven R, Vatten LJ (2006). Increased risk of placenta previa in pregnancies following IVF/ICSI; a comparison of ART and non-ART pregnancies in the same mother. Hum Reprod.

[5] De Sutter P, Veldeman L, Kok P, Szymczak N, Van der Elst J, Dhont M (2005). Comparison of outcome of pregnancy after intra-uterine insemination (IUI) and IVF. Hum Reprod.

[6] Poikkeus P, Gissler M, Unkila-Kallio L, Hyden-Granskog C, Tiitinen A (2007). Obstetric and neonatal outcome after single embryo transfer. Hum Reprod.

[7] Göçmen A, Güven Ş, Bağci S, Çekmez Y, Şanlıkan F (2015). Comparison of maternal and fetal outcomes of IVF and spontaneously conceived twin pregnancies: three year experience of a tertiary hospital. Int J Clin Exp Med.

[8] National High Blood Pressure Education Program Working Group on High Blood Pressure in Pregnancy (2000). Report of the national high blood pressure education program working group on high blood pressure in pregnancy. Am J Obstet Gynecol.

[9] Magee LA, Helewa M, Rey E (2008). Hypertension guideline committee; strategic training initiative in research in the reproductive health sciences (stirrhs) scholars Diagnosis evaluation and management of the hypertensive disorders of pregnancy. J Obstet Gynaecol Can.

[10] American college of obstetricians and gynecologists, task force on hypertension in pregnancy (2013). Hypertension in pregnancy. Report of the american college of obstetricians and gynecologists’ task force on hypertension in pregnancy.

[11] Tebeu PM, Foumane P, Mbu R, Fosso G, Biyaga PT, Fomulu JN (2011). Risk factors for hypertensive disorders in pregnancy: a report from the maroua regional hospital, cameroon. J Reprod Infertil.

[12] Raymond D, Peterson E (2011). A critical review of early-onset and late-onset preeclampsia. Obstet Gynecol Surv.

[13] Thomopoulos C, Tsioufis C, Michalopoulou H, Makris T, Papademetriou V, Stefanadis C (2013). Assisted reproductive technology and pregnancy-related hypertensive complications: a systematic review. J Hum Hypertens.

[14] American college of obstetricians and gynecologists (2012). ACOG practice bulletin no 125: chronic hypertension in pregnancy. Obstet Gynecol.

[15] Bramham K, Parnell B, Nelson-Piercy C, Seed PT, Poston L, Chappell LC (2014). Chronic hypertension and pregnancy outcomes: systematic review and meta-analysis. BMJ.

[16] Backes CH, Markham K, Moorehead P, Cordero L, Nankervis CA, Giannone PJ (2011). Maternal preeclampsia and neonatal outcomes. J Pregnancy.

[17] Watanabe N, Fujiwara T, Suzuki T, Jwa SC, Taniguchi K, Yamanobe Y (2014). Is in vitro fertilization associated with preeclampsia? A propensity score matched study. BMC Pregnancy Childbirth.

[18] von Elm E, Altman DG, Egger M, Pocock SJ, Gøtzsche PC, Vandenbroucke JP (2008). STROBE Initiative The strengthening the reporting of observational studies in epidemiology (strobe) statement: guidelines for reporting observational studies. J Clin Epidemiol.

[19] Poon LC, Kametas NA, Chelemen T, Leal A, Nicolaides KH (2010). Maternal risk factors for hypertensive disorders in pregnancy: a multivariate approach. J Hum Hypertens.

[20] Kosovo Unemployment Rate 2001-2016.

[21] Davalos ME, Sattar S, Simler K, Bidani B, Reva A, Tsirunyan S, Orlando MB (2012). Kosovo - Gender gaps in education, health, and economic opportunities. The World Bank Group Europe and Central Asia region poverty reduction and economic management.

[22] Sullivan EA, Chapman MG, Wang YA, Adamson GD (2010). Population-based study of cesarean section after in vitro fertilization in Australia. Birth.

[23] Gillet E, Martens E, Martens G, Cammu H (2011). Prelabour caesarean section following IVF/ICSI in older-term nulliparous women: too precious to push?. J Pregnancy.

